# Brain Stroke Classification via Machine Learning Algorithms Trained with a Linearized Scattering Operator

**DOI:** 10.3390/diagnostics13010023

**Published:** 2022-12-21

**Authors:** Valeria Mariano, Jorge A. Tobon Vasquez, Mario R. Casu, Francesca Vipiana

**Affiliations:** Department of Electronics and Telecommunications, Politecnico di Torino, 10129 Torino, Italy

**Keywords:** microwave imaging, machine learning algorithms, support vector machines, multilayer perceptrons, k-nearest neighbours, brain stroke

## Abstract

This paper proposes an efficient and fast method to create large datasets for machine learning algorithms applied to brain stroke classification via microwave imaging systems. The proposed method is based on the distorted Born approximation and linearization of the scattering operator, in order to minimize the time to generate the large datasets needed to train the machine learning algorithms. The method is then applied to a microwave imaging system, which consists of twenty-four antennas conformal to the upper part of the head, realized with a 3D anthropomorphic multi-tissue model. Each antenna acts as a transmitter and receiver, and the working frequency is 1 GHz. The data are elaborated with three machine learning algorithms: support vector machine, multilayer perceptron, and k-nearest neighbours, comparing their performance. All classifiers can identify the presence or absence of the stroke, the kind of stroke (haemorrhagic or ischemic), and its position within the brain. The trained algorithms were tested with datasets generated via full-wave simulations of the overall system, considering also slightly modified antennas and limiting the data acquisition to amplitude only. The obtained results are promising for a possible real-time brain stroke classification.

## 1. Introduction

Brain stroke is a cardiovascular disease that occurs when the blood flow becomes abnormal in a region of the head. It is one of the main causes of death and disability. There are two types of stroke: ischemic and haemorrhagic. The first one is the most common and occurs when a brain vessel is clogged; the second one is the most deadly and happens when there is bleeding. They have two different medical treatments to be applied right after the stroke onset, and mistaking them could be fatal for the patient [1]. Currently, clinicians rely on two well-established imaging techniques to identify the stroke location and kind: magnetic resonance imaging (MRI) and computerized tomography (CT). These techniques have a good resolution, but the corresponding imaging systems are time-consuming, expensive, not portable, and, in the CT case, harmful. Hence, in the last few years, other imaging techniques have been studied, and in particular, microwave imaging (MWI) has shown very promising capabilities for brain stroke imaging [2]. The basic concept of MWI is that, at microwave frequencies, there is a dielectric contrast between the healthy brain tissues and the ischemic or haemorrhagic stroke area. In particular, the dielectric permittivity of ischemic stroke is lower than the permittivity of the surrounding brain tissues; instead, the dielectric permittivity of haemorrhagic stroke (i.e., the blood permittivity) is higher than the permittivity of brain tissues. An MWI system consists of an antenna array, placed around the head, where, in general, each antenna acts as a transmitter and receiver. The received data are, then, elaborated by an imaging algorithm to locate the stroke and identify its kind [3,4]. MWI devices are portable, low cost, and safe, because they use low-power microwave radiations, but the imaging algorithms, which elaborate the measured data, can be time-consuming, not allowing a real-time brain stroke classification.

Machine learning (ML) algorithms represent an innovative approach, recently entered in the world of biomedical imaging: they can be an interesting alternative to deterministic imaging algorithms, reducing significantly the data elaboration time [5]. The first pioneering microwave device, implementing ML-based classification techniques, was proposed in [6] for stroke type detection in the pre-hospital phase, with the aim of reducing the intervention time. Instead, in [7], Salucci and co-authors realized a stroke classification in steps, following the clinical diagnosis process (i.e., detection, identification, and localization) through the support vector machine algorithm. In [8], a portable microwave-based diagnostic system for internal haemorrhage was proposed, which exploits the deep neural network inference, while, in [9], brain stroke types were classified implementing the complex network method.

However, the use of ML algorithms, for brain stroke classification, can have an important drawback: a very large dataset is needed to properly train the ML algorithms. The dataset can be generated with clinical data, laboratory measurements with anthropomorphic head phantoms, or with electromagnetic (EM) simulations of the overall system. However, collecting a large number of clinical data can be difficult, and several measurements, with different head conditions (healthy and in the presence of the stroke), as well as full-wave simulations require significant execution time. In order to overcome this limit, in [10], the classification was made with an unsupervised method exploiting the brain symmetry, while, in [11], the classification was based on the signals’ correlation in a transmission line without the need for a training phase. The aim of this work is to propose an alternative and efficient method, based on the distorted Born approximation and the linearization of the scattering operator, in order to generate the training dataset. The obtained dataset generation time, for each head condition, is around four orders of magnitude lower, with negligible computational requirements, with respect to standard full-wave simulations of the overall MWI system (i.e., few seconds versus hours). The proposed method was applied to the training of three ML algorithms: support vector machine (SVM), multilayer perceptron (MLP) belonging to the family of neural network algorithms, and k-nearest neighbours (k-NN). To the best of the authors’ knowledge, the k-NN algorithm is here exploited, for the first time, in brain stroke detection with a microwave imaging system. The ML testing was, then, performed through data generated via full-wave simulations of the overall MWI system applied to a 3D anthropomorphic multi-tissue head model. The testing data were also generated slightly modifying the antennas’ geometry (as could happen in the antenna realization phase) and limiting to the data amplitude, i.e., discarding the phase. In most cases, the tested ML algorithms are able to accurately detect the presence of the stroke, its kind, and its location inside the brain. Moreover, different multi-tissue head models were generated starting from the original one, and they were inserted in the training set and in the testing set in order to assess the capability of the algorithms to generalize and correctly classify also samples with different head models. In this case, the SVM and MLP algorithms obtained positive results; in fact, they were able to precisely classify samples of the testing set related to head models that were not present in the training set. Preliminary analyses with just an homogeneous head model were recently presented in [12,13,14].

The paper is organized as follows. Section 2 reports a general description of the MWI system. In Section 3, there is a brief presentation of the ML algorithms exploited to classify our dataset. Section 4 describes the proposed method to generate the training set and how the overall system is tested. Numerical results are presented in Section 5, and finally, conclusions and perspectives are given in Section 6.

## 2. Microwave Imaging System and Head Model

**Figure 1:**
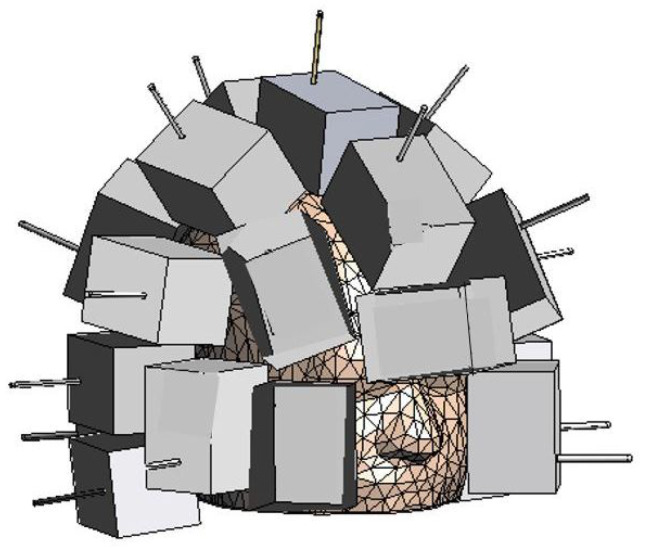
CAD model of the 3D MWI system together with the head phantom.

The used MWI system, presented in [15], is shown in Figure 1. It consists of $M_{a}=24$ antennas, acting as both transmitters and receivers, placed conformally to the upper part of the head, like a “helmet”. The number of antennas, spatial distribution, and orientation were obtained following the design process proposed in [16], which allows maintaining good imaging performance with a low system complexity. In particular, Reference [16] analysed the scattering operator, focusing on its discretized version and condition number, taking into account the actual dynamics and signal-to-noise ratio of the measurement system. If the number of antennas is kept small, the reconstruction is more stable (i.e., the scattering operator condition number is low), but it will translate into a reduced reconstruction accuracy, due to the loss in terms of collected information. However, too many antennas will collect additional information that cannot be accurately reconstructed due to the finite dynamic range of the system. Hence, the optimal final configuration is a trade-off between the reconstruction accuracy and the stability of the problem, as detailed in [16].

Then, each antenna is placed inside a dielectric “brick”, which works as the coupling medium in order to maximize the field penetration within the head [17]: it is inconvenient to place the antennas in air because the significant difference between air’s and head tissues’ permittivity, which would generate a strong field reflection at the air–head interface. Moreover, the dielectric permittivity of the coupling medium and the working frequency were chosen in order to obtain a good balance between imaging resolution and wave penetration; in fact, the high frequencies lead to better resolution, but the penetration depth of the signal decreases. In [18], there was a study of the transmission coefficient as a function of the frequencies and permittivity of the coupling medium. In particular, considering the permittivity, thickness, and order of the different tissues in the head, there is a forbidden frequency band in the 1.5–4 GHz range in which the transmission coefficient is lower. Keeping in mind these considerations, the chosen working frequency was 1 GHz with a coupling medium permittivity of around 20.

**Figure 2:**
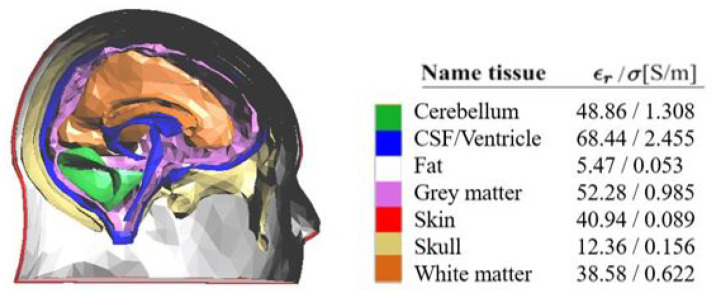
Left: sagittal cut of the used 3D multi-tissue head phantom; right: dielectric properties of all the head tissues at 1 GHz.

The head was modelled with a 3D anthropomorphic phantom available in [19,20]: a sagittal cut is shown in Figure 2, together with the relative permittivity, $\epsilon_{r}$, and conductivity, σ, of all the different tissues at 1 GHz. This model represents the considered reference healthy human head.

## 3. Machine Learning Algorithms

In this section, we give a brief description of the ML algorithms used to classify the dataset. All the algorithms exploit the supervised method: they create a model based on the training set, in which the examples are labelled each with their own specific class. When they receive as input the attributes of the test data, they should be able to return the correct class. In this work, the classes are the presence, the type (ischemic or haemorrhagic), and the position of the stroke within the brain.

The first algorithm is SVM, which works with an optimization method, in which the first step is to map the training dataset into a higher-dimensional space. In the new space, SVM finds the hyperplane able to optimally separate the records belonging to different classes. The algorithm chooses the hyperplane with the highest distance to the closest training record of each class [21,22].

The MLP algorithm is an artificial neural network, inspired by the human biological neural network. The basic element of the MLP is the “neuron”, linked to the others by weighted connections. The neuron output is regulated by an activation function. During the training process, the model modifies the weights in order to train the network to produce useful output. An MLP is composed by layers of neurons: one input layer, one output layer, and, in the middle, at least one hidden layer.

In this work, the feed-forward network is exploited: the output of a layer is the input of the next layer; hence, there are no loops [23]. In [24], this algorithm was successfully applied by the authors to a microwave imaging system designed for detecting contaminants in packaged food products moving along the conveyor belt.

The k-NN algorithm is one of the easiest learning algorithms. It is based on the similarity principle: for each testing example, it calculates the distance from all training examples in a multidimensional space. The distance usually is the Euclidean one, but there are no restrictions. Then, the k-NN assigns to the test case the more frequent class among the *k* training examples closer to the considered test case [25].

The ML algorithms were implemented in Python on Google Colab platform [26], using the open-source library scikit-learn explained in [27] and available in [28]. The chosen parameters for each ML algorithm are detailed in Section 5.

## 4. Training Set Generation and Testing Procedure

**Figure 3:**
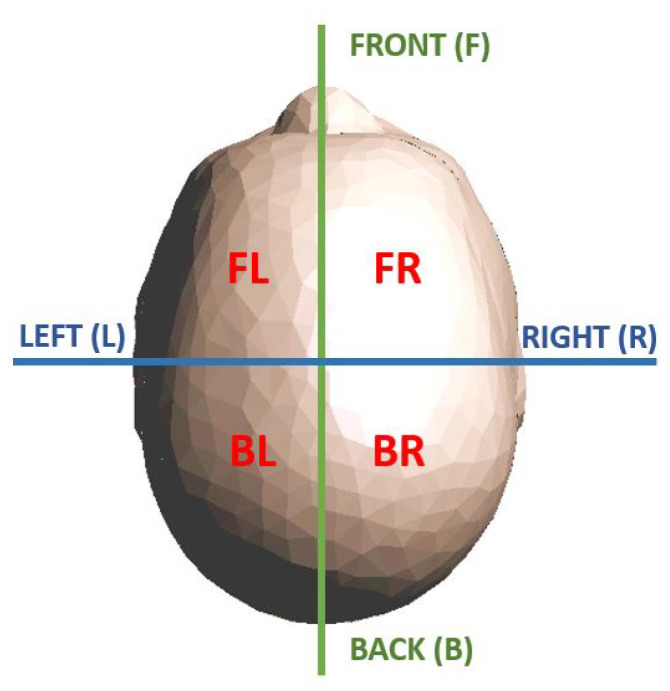
Subdivision of the head into four regions (horizontal view): front left (FL), front right (FR), back left (BL), and back right (BR).

In the following, first, the linearized scattering operator is derived and discretized (Section 4.1) and, then, used to generate the proposed training dataset (Section 4.2). The dataset contains nine classes differentiated for presence (or absence), typology (ischemic or haemorrhagic), and position (four different head regions) of the stroke within the brain. In order to classify the stroke location, the brain is divided into four regions, as shown in Figure 3. Finally, in Section 4.3, the testing procedure is detailed, where the whole MWI system, together with the 3D anthropomorphic multi-tissue head model, is simulated with full-wave simulations.

### 4.1. Linearized Scattering Operator

The considered domain of interest (DoI) is the whole head, with all the different tissues characterized by the corresponding dielectric properties, as shown in Figure 2. Then, the dielectric contrast can be defined as:

(1) $$\Delta \chi \left(\underline{r}\right)\overset{\Delta }{=}\frac{\epsilon_{s}\left(\underline{r}\right)-\epsilon_{b}\left(\underline{r}\right)}{\epsilon_{b}\left(\underline{r}\right)},$$

where $\underline{r}$ is a generic point in the DoI and $\epsilon_{b}\left(\underline{r}\right)$ and $\epsilon_{s}\left(\underline{r}\right)$ are the complex permittivities, located in $\underline{r}$, of the corresponding head tissue and of the (ischemic or haemorrhagic) stroke, respectively. If the stroke is not present in $\underline{r}$, $\epsilon_{s}\left(\underline{r}\right)$ is equal to $\epsilon_{b}\left(\underline{r}\right)$, and consequently, $\Delta \chi (\underline{r})=0$.

With the MWI system described in Section 2, it is possible to evaluate the scattering parameters $S_{p,q}$ between all antenna pairs at the antenna ports, where *p* is the receiver and *q* the transmitter, with $p,q=1,\ldots ,M_{a}$. Then, for each p,q antenna pair, we can define the corresponding *differential* scattering parameter as

(2) $$\Delta S_{p,q}=S_{p,q}^{t}-S_{p,q}^{b},$$

with $p,q=1,\ldots ,M_{a}$, where $S_{p,q}^{t}$ and $S_{p,q}^{b}$ are the scattering parameters evaluated in the scenario under test and in the background scenario (i.e., the reference healthy head), respectively. Each p,q differential scattering parameter is related to the dielectric contrast [29,30] as

(3) $$\Delta S_{p,q}=-\frac{j\;\omega \;\epsilon_{b}\left(\underline{r}\right)}{2\;a_{p}\;a_{q}}\int \int \int_{V}{\underline{E}}_{p}^{t}\left(\underline{r}\right)\cdot {\underline{E}}_{q}^{b}\left(\underline{r}\right)\;\Delta \chi \left(\underline{r}\right)\;d^{3}\underline{r},$$

where *V* is the DoI volume, ω is the angular frequency, and $a_{p}$ and $a_{q}$ are the power waves at the p,q antennas’ ports, respectively. The ${\underline{E}}_{p}^{t}\left(\underline{r}\right)$ and ${\underline{E}}_{q}^{b}\left(\underline{r}\right)$ vectors are the electric field radiated, in the point $\underline{r}$ of the DoI, by the *p* antenna in the case of the scenario under test and by the *q* one for the background scenario, respectively.

If there is a limited variation between the scenario under test and the background one, as, e.g., at the initial phases of the stroke onset, the distorted Born approximation can be applied to (3) assuming that ${\underline{E}}_{p}^{t}\left(\underline{r}\right)≅{\underline{E}}_{q}^{b}\left(\underline{r}\right)$. Hence, the relation between each p,q differential scattering and the dielectric contrast can be written as

(4) $$\Delta S_{p,q}≅S\left\{\Delta \chi (\underline{r})\right\},$$

where S is now a *linear* and compact integral operator, called in the following scattering operator, a function of the electric field radiated by the *p* and *q* antennas in the background scenario only.

As in [15], the DoI can be discretized with *N* tetrahedral cells, associating with each tetrahedron the complex permittivity of the corresponding discretized tissue. Applying this discretization procedure to (4), we obtain

(5) $$\left[\Delta S\right]≅\left[S\right]\left[\Delta \chi \right],$$

where $\left[\Delta S\right]$ is an M×1 array collecting all the differential scattering parameters, with $M=M_{a}^{2}$, and $\left[\Delta \chi \right]$ is an N×1 array collecting all the dielectric contrast values associated with each tetrahedron. The M×N matrix $\left[S\right]$ represents the *discretized* scattering operator, where each m,n element is equal to

(6) $${\left[S\right]}_{m,n}=K_{m,n}\;{\underline{E}}_{p}^{b}\left({\underline{r}}_{n}\right)\cdot {\underline{E}}_{q}^{b}\left({\underline{r}}_{n}\right)\;\Delta V_{n},$$

where the index *m* corresponds to the indices’ pair (p,q), $K_{m,n}=\left[-j\;\omega \;\epsilon_{b}\left({\underline{r}}_{n}\right)/\left(2\;a_{p}\;a_{q}\right)\right]$, ${\underline{r}}_{n}$ is the barycentre of the *n* tetrahedron, and $\Delta V_{n}$ its volume. Applying (5), all the differential scattering parameters for the considered scenario under test, $\left[\Delta S\right]$, can be easily evaluated by just multiplying the matrix $\left[S\right]$ by the vector $\left[\Delta \chi \right]$, representing the corresponding dielectric contrast. Moreover, the matrix $\left[S\right]$ depends on the background scenario only, and therefore, it has to be evaluated and stored only one time. Here, the fields ${\underline{E}}_{p}^{b}\left({\underline{r}}_{n}\right)$ and ${\underline{E}}_{q}^{b}\left({\underline{r}}_{n}\right)$ in (6) are obtained via an in-house full-wave (FEM) solver [31,32].

Finally, the scattering parameters of the scenario under test are obtained by just adding the corresponding scattering parameters of the background scenario to the differential scattering parameters as in (2). As for the discretized scattering operator, also the scattering parameters of the background scenario have to be evaluated and stored one time only. It is evident that the proposed procedure to evaluate the scattering parameters of the scenario under test is much faster than a procedure using full-wave simulations, which have to be repeated for each considered scenario used to train the ML algorithm. The time reduction is around four orders of magnitude with respect to FEM full-wave simulations [31].

### 4.2. Training Set Generation

**Figure 4:**
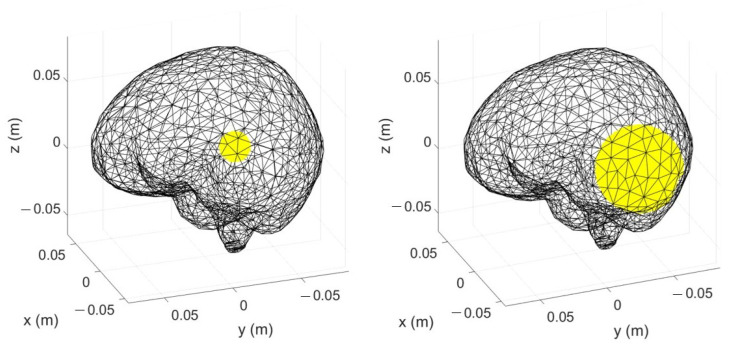
Discretized brain together with spherical strokes (in yellow); left: the smallest stroke size (radius =1 cm and volume =4.2 cm${}^{3}$); right: the largest stroke size (radius =3 cm and volume =113.1 cm${}^{3}$).

The first step to generate the dataset to train the ML algorithm is the $\left[\Delta S\right]$ generation for different scenarios, as in (5). In each considered scenario, a stroke, with a spherical shape, is inserted inside the multi-tissue head model (see Figure 2). The strokes can have five possible radii, from 1 to 3 cm, with a step of 0.5 cm, which correspond to a volume between 4.2 cm${}^{3}$ and 113.1 cm${}^{3}$, as shown in Figure 4.

In real life, the stroke volume depends on many factors, such as the interested blood vessels, the stroke location, and the diagnosis time (see, e.g., [33,34,35,36]), and it can be estimated from about 2 to 200 cm${}^{3}$: this range includes the stroke volume variation here considered. The chosen lower bound of the stroke radius (i.e., 1 cm) corresponds to the minimum resolution of the used MWI system [31], while the upper bound (i.e., 3 cm) corresponds to the maximum stroke size, which allows an acceptable error between (3) and (4) and, so, justifies the use of the linearized scattering operator in the generation of the training sets.

**Figure 5:**
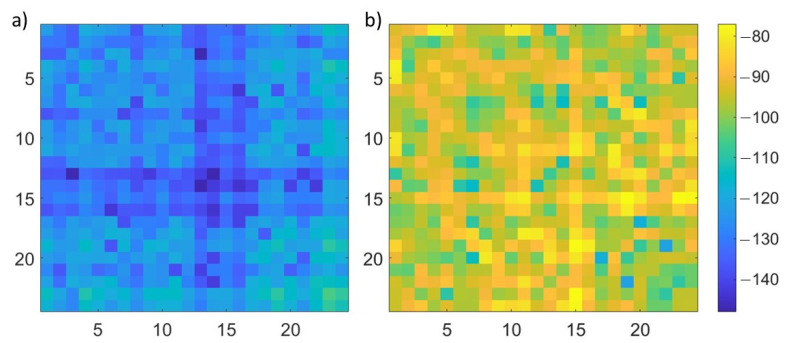
$\eta_{p,q}$ error, for $p,q=1,\ldots ,M_{a}$, dB units; (**a**) 1 cm stroke radius, (**b**) 3 cm stroke radius.

**Figure 6:**
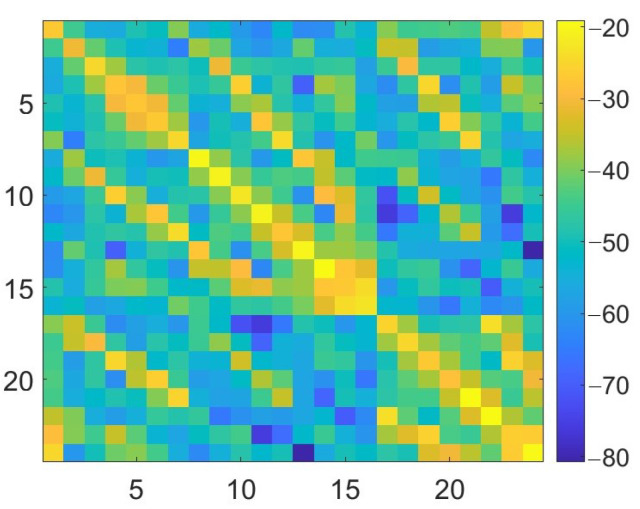
Amplitude of $S_{p,q}^{\mathrm{FW}}$ for $p,q=1,\ldots ,M_{a}$, dB units in a simulation with a 3 cm stroke radius.

This error can be defined as

(7) $$\eta_{p,q}=20\log_{10}\left(\left|S_{p,q}^{\mathrm{FW}}-S_{p,q}^{L}\right|\right),$$

where $S_{p,q}^{\mathrm{FW}}$ and $S_{p,q}^{L}$ are the scattering parameters, for each p,q antennas’ pair, for the scenario under test, obtained with the full-wave simulations and through the linearized scattering operator, respectively. In Figure 5, the $\eta_{p,q}$ error, for $p,q=1,\ldots ,M_{a}$, is shown for the two extremes of the considered stroke radius range: 1 cm (on the left) and 3 cm (on the right). As expected, the error for the 3 cm stroke is higher than the one for the 1 cm stroke, but the highest error values, equal to around −80 dB, are comparable with the smallest values of the $S_{p,q}^{\mathrm{FW}}$ (full-wave simulation with a 3 cm stroke radius), $p,q=1,\ldots ,M_{a}$, shown in Figure 6.

The centre of each stroke is chosen randomly among the tetrahedra barycentres inside the brain, which includes the white matter, grey matter, and cerebellum. To avoid each time a new discretization of the whole head model, the stroke is simply obtained by collecting all the tetrahedra with a distance from the stroke centre lower than the stroke radius. If some selected tetrahedra are outside the brain (i.e., outside the white matter, grey matter, and cerebellum), they are simply discarded, while, if more than half of the selected tetrahedra fall outside the brain, that stroke case is discarded.

The dielectric characteristics of the ischemic stroke are $\epsilon_{r,isch}=36.00$ and $\sigma_{isch}=0.72$ S/m, at 1 GHz. The minimum dielectric contrast module is 0.08 and the maximum is 0.31 with respect to the white matter and grey matter, respectively. Instead, the haemorrhagic stroke has $\epsilon_{r,blood}=63.41$ and $\sigma_{blood}=1.58$ S/m with a minimum dielectric contrast module of 0.28 and a maximum of 0.75 with respect to the grey matter and white matter, respectively [37].

The generated scattering parameters are contaminated with noise. We selected four different noise levels for them: −110 dB, −105 dB, −95 dB, and −90 dB. The minimum chosen noise level is comparable with the noise floor of a medium-quality vector network analyser [38], while the maximum noise level is up to 20 dB higher than the noise floor. Assuming that the noise is due to random variations in the dielectric contrast (a consequence of differences in the tissues’ dielectric properties in the considered scenarios), we first generated variations in the dielectric contrast domain and, then, through

(8) $$\left[\delta S\right]≅\left[S\right]\left[\delta \chi \right]$$

we obtained the corresponding noise in the scattering parameters. In (8), $\left[\delta S\right]$ is an M × 1 array, which collects the corresponding noise in the scattering parameters space, while $\left[\delta \chi \right]$ is an N × 1 array, collecting the dielectric contrast random noise associated with each tetrahedron.

**Figure 7:**
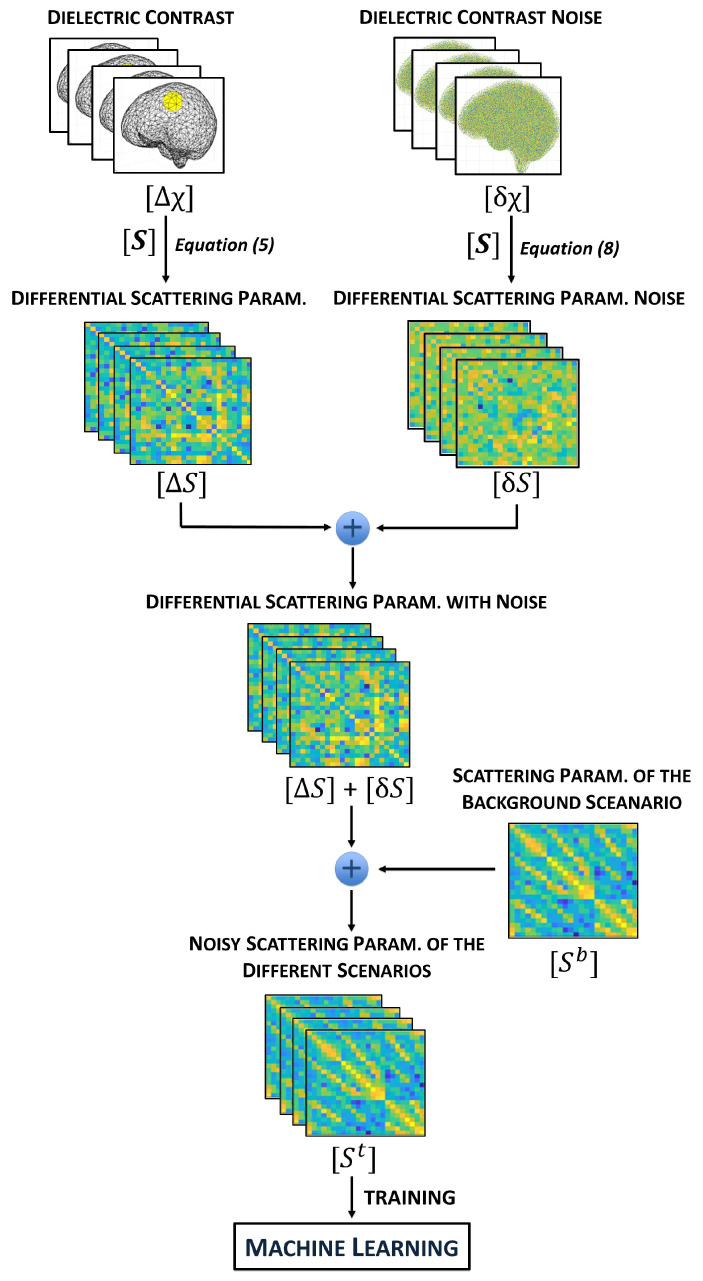
Flow-chart of the procedure for the training set generation.

During the training set generation, for each case, the noise δχ was chosen randomly among the selected four levels. Then, δχ was added to the created dielectric contrast in the DoI, Δχ, and the corresponding differential scattering parameters, ΔS (now corrupted by noise), were generated via (5). Finally, the scattering parameters of the background scenario, $S^{b}$, were summed to ΔS in order to find the scattering parameters in the scenario under test, $S^{t}$, which forms the training set of the ML algorithm. We summarize the whole procedure in the flow-chart in Figure 7.

The final training set contains 10,000 records, almost equally distributed among the nine classes: 1000 for the class of data without stroke presence, labelled in the following with “N”, and 1125 for all the other classes, labelled with “I” for ischemic strokes and “H” for haemorrhagic ones, followed by the position name, “FL” front left, “FR” front right, “BL” back left, or “BR” back right, as described in Figure 3.

Due to the reciprocity of the MWI system, only the independent data are given in the input to the ML algorithm, corresponding to 300 complex elements (superior triangular of the 24 × 24 S parameters matrix). Moreover, for each considered element, the real and imaginary parts are exploited as two different dataset features. Therefore, the dataset has 600 features.

The total time needed to generate the final training set was almost 13 h via a non-optimized Matlab code running on a server Intel Xeon Dilver 4116 CPU @ 2.10 GHz with a memory of 767 GB and 24 cores. The estimated time to generate the same training set via FEM full-wave simulations is almost 3.5 years, making it clearly unfeasible.

### 4.3. Testing Procedure

**Figure 8:**
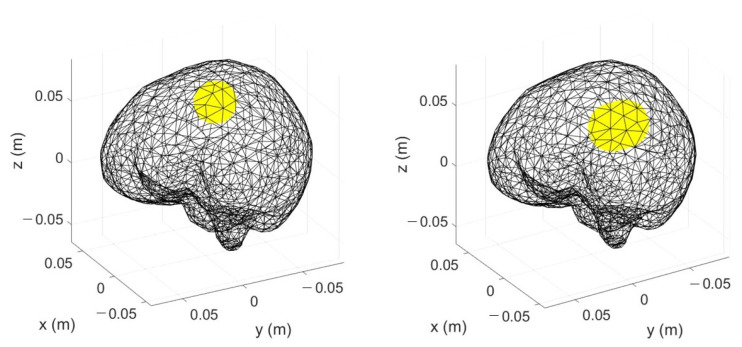
Discretized brain together with strokes (in yellow); left: spherical shape (volume = 14.14 cm${}^{3}$); right: ellipsoid shape (volume = 30.57 cm${}^{3}$).

To generate the testing set, first, the scenario under analysis together with the MWI system was simulated with a full-wave FEM solver as in [31] in order to evaluate the corresponding scattering parameters at the antenna ports. The scenario can be the healthy one, or it can contain a ischemic or haemorrhagic stroke. The stroke shape can be spherical or ellipsoidal, and its volume is within the range used for the training set. Two examples of strokes used in the testing set are shown in Figure 8.

**Figure 9:**
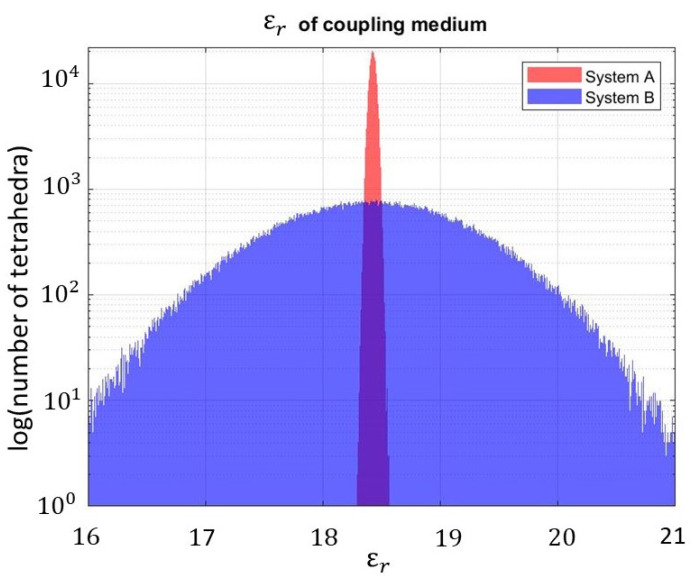
Testing procedure: number of tetrahedra in the dielectric bricks with respect to the associated $\epsilon_{r}$; red: standard deviation equal to 0.03; blue: standard deviation equal to 2.

Observing that the geometry and the materials of the MWI device can be not fully known, the full-wave simulated MWI system, used in the testing phase, is not identical to the one simulated to generate the discretized scattering operator in the training phase. In particular, here, we considered variations in the dielectric characteristics of the bricks (i.e., in the coupling medium around the antennas). The dielectric bricks can be obtained through a mixture of graphite powder and urethane rubber, which can have some inaccuracies in the obtained relative permittivity with respect to the nominal one, equal to 18.42 [17]. Hence, in the training phase, all the tetrahedra representing the dielectric bricks have an associated relative permittivity equal to the nominal one, while, in the testing phase, the relative permittivity associated with these tetrahedra changes as a normal distribution with the mean equal to the nominal relative permittivity. Two families of non-nominal systems were considered: “System A” with the standard deviation equal to 0.03 (very close to the nominal case) and “System B” with the standard deviation equal to 2.00, as reported in Figure 9.

**Figure 10:**
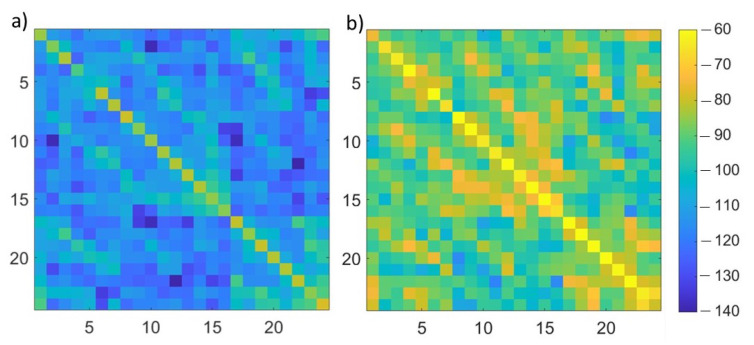
Difference between the scattering parameters for Systems A (**a**) and B (**b**) with respect to the nominal system, dB units.

In Figure 10, the difference between the scattering parameters for Systems A (left) and B (right) with respect to the nominal system is reported. This difference is evaluated as in (7). As expected, the difference is higher in System B, where there is a higher variation of the dielectric brick permittivity with respect to the nominal one.

Then, the noise, obtained via (8), is added to each set of scattering parameters. Finally, the noisy scattering parameters are calibrated to the nominal system [39,40] as follows:

(9) $${\tilde{S}}_{p,q}^{t,C}=\frac{S_{p,q}^{b}}{\left({\tilde{S}}_{p,q}^{b}+\delta S_{1}\right)}\cdot \left({\tilde{S}}_{p,q}^{t}+\delta S_{2}\right),$$

where, for each p,q antennas’ pairs, $S_{p,q}^{b}$ are the scattering parameters of the background scenario using the nominal system, while ${\tilde{S}}_{p,q}^{b}$ and ${\tilde{S}}_{p,q}^{t}$ are the scattering parameters, using System A or B, of the the background scenario and of the scenario under test, respectively. $\delta S_{1}$ and $\delta S_{2}$ represent the added noise. The obtained ${\tilde{S}}_{p,q}^{t,C}$ sets, for $p,q=1,\ldots ,M_{a}$, are used to test the trained ML algorithms.

## 5. Numerical Results

In this section, the results, obtained with the three selected ML algorithms (i.e., MLP, SVM, and k-NN; see Section 3) are described in detail.

We start with a validation, where 80% of the records, created with the linear scattering operator (see Section 4.1), were used as training set (i.e., 8000 records), while 20% represents the validation set (i.e., 2000 records).

Then, the testing was performed with the scattering parameters generated with the full-wave simulations of Systems A and B (see Section 4.3), given in the input to the the ML algorithms, trained with the 8.000 records generated via the linear scattering operator (see Section 4.2). The generated records for the testing were 125 for each system: 10 records belong to the class “N” (i.e., healthy scenario), 60 to the ischemic stroke classes, and 55 to the haemorrhagic stroke ones. In particular, among the ischemic records, 2 cases are in the class “I_FL” (2 cm radially spherical strokes), 3 cases in the class “I_FR” (1 cm, 1.5 cm, and 2.5 cm radially spherical strokes), 3 cases in the class “I_BL” (2 spherical strokes of 1 cm in radius and 1 ellipsoid stroke with 0.75×1×1 cm axes), and 4 cases in the class “I_BR” (1 spherical stroke of 1.5 cm in radius and 3 of 2.5 cm in radius). Instead, among the haemorrhagic stroke classes, 4 cases are in the class “H_FL” (2 spherical strokes of 1 cm in radius, 1 of 1.5 cm in radius, and 1 of 2.5 cm in radius), 3 cases in the class “H_FR” (1 spherical stroke of 1 cm in radius and 1 of 2 cm in radius), 3 cases in the class “H_BL” (2 spherical strokes of 1.5 cm and 2.5 cm in radius and 1 ellipsoid stroke with with 0.75×1×1.5 cm axes), 2 cases in the class “H_BR” (3 cm in radius spherical strokes). Finally, both the ischemic and haemorrhagic stroke records were extended by a factor five by adding different noises (see Section 4.3).

The grid search method [41] was used here to find the best set of hyperparameters for each ML algorithm. Different values of the hyperparameters were given in the input to the grid search method, which builds a model for each hyperparameter combination. Finally, it chooses the set with the best score, that is, as in our case, the model accuracy, defined as the ratio of correctly predicted samples to the total number of samples.

In the following, the ML algorithms’ performances are evaluated through the “confusion matrix”. The confusion matrix is a square matrix with dimension equal to the number of classes, in our case nine classes. This matrix has, on the rows, the true classes and, on the columns, the predicted classes, so that the records in the main diagonal are correctly predicted. For both the validation and the testing, we report the confusion matrices with the exact number of records in each matrix element and the normalized confusion matrices where each row is normalized with respect to the total number of class records. In this way, it is evident, for each class, the percentage of records correctly or wrongly classified. Moreover, on the reported confusion matrices, three squares are highlighted to identify macro-classes: the healthy scenario (in yellow), the ischemic stroke (in green), and the haemorrhagic stroke (in red).

In Section 5.1, the ML algorithms have as features the real and imaginary parts of the scattering parameters, while, in Section 5.2, we try to limit them to the amplitude only. The amplitude and phase of the scattering parameters were been also tried as features, and the results were identical to the real and imaginary parts’ case; hence, they are not reported in the following. Finally, in Section 5.3, we introduce more head models in the training set, in order to simulate not only different individuals, but also different positions of the same head in the system. Moreover, in the same section, we explain how head models different from those of the training set are used in the training set, in order to verify the generalization capabilities of the algorithms.

### 5.1. Complex Datasets

Through the grid search method, the set of hyperparameters was selected for each ML algorithm. For SVM, the kernel used was the radial basis function with the kernel coefficient γ equal to 0.059 and the penalty parameter *C* equal to 300. For MLP, the activation function was the hyperbolic tangent, with five hidden layers with 800, 400, 200, 100, and 50 neurons each, the solver was the stochastic gradient descent, the regularization term α was equal to 0.0001, both the maximum number of epochs and the size of the batch were equal to 200, and it used an adaptive learning rate. Finally, for the k-NN, the distance metric was the Euclidean one, with *k* equal to 3.

**Figure 11:**
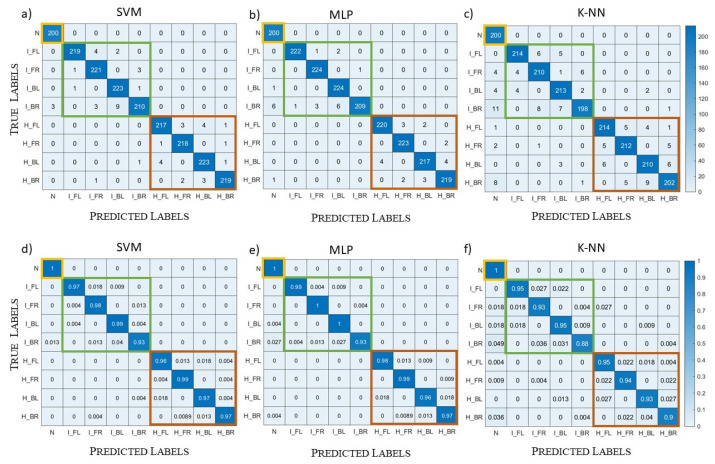
Validation case, complex datasets: (**a**–**c**) confusion matrices; (**d**–**f**) normalized confusion matrices; (**a**,**d**) SVM results; (**b**,**e**) MLP results; (**c**,**f**) k-NN results.

Figure 11 shows the confusion matrices obtained with the three algorithms in the validation; the first row contains the confusion matrices with the exact number of records, while the second row contains the normalized confusion matrices. Moreover, the first column corresponds to the SVM results, the second one to the MLP results, and the last one to the k-NN results. All the records belonging to the class “N” (i.e., healthy scenario) were correctly classified by all three algorithms. Then, the results of the other classes were similar between SVM, reported in Figure 11a,d, and MLP, reported in Figure 11b,e. Indeed, SVM did not correctly classify 2.4% of the total records in the validation set and MLP 2.1%. Instead, k-NN, due to its operating mechanism, seems more sensitive to noise, and it classified more ischemic and haemorrhagic samples as healthy with respect to the other two algorithms, as shown in Figure 11c,f. Its percentage of badly classified records was equal to 4.3%, which is worse than the other two algorithms. Moreover, analysing k-NN, records wrongly classified as healthy correspond to the most critical cases with the highest noise level (i.e., −90 dB) and the lowest stroke dimension (i.e, 1 cm-radius sphere).

**Figure 12:**
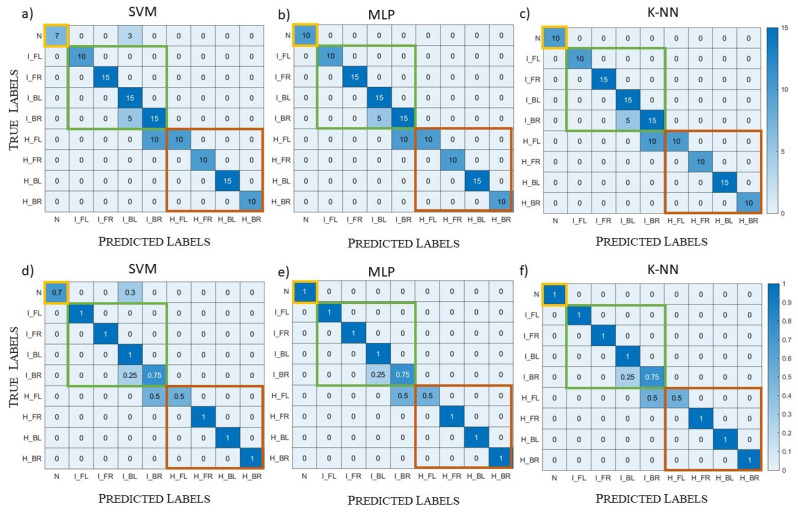
System A testing case, complex datasets: (**a**–**c**) confusion matrices; (**d**–**f**) normalized confusion matrices; (**a**,**d**) SVM results; (**b**,**e**) MLP results; (**c**,**f**) k-NN results.

**Figure 13:**
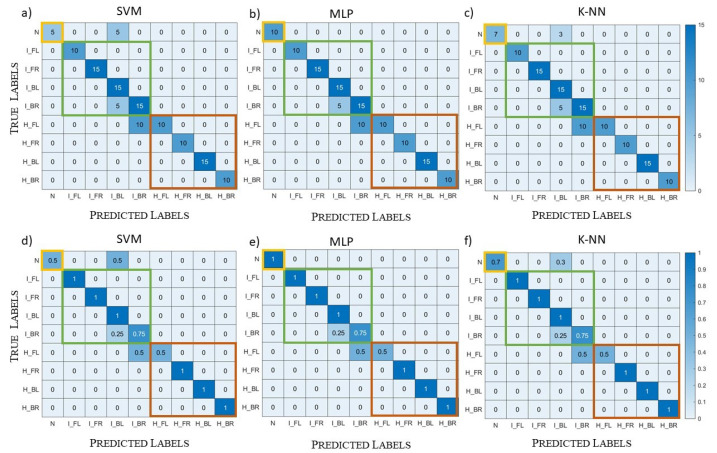
System B testing case, complex datasets: (**a**–**c**) confusion matrices; (**d**–**f**) normalized confusion matrices; (**a**,**d**) SVM results; (**b**,**e**): MLP results; (**c**,**f**) k-NN results.

The results for the testing of Systems A and B are reported in Figure 12 and Figure 13, respectively. For System A, both MLP and k-NN never missed the macro-classes, highlighted with yellow, green, and red squares in Figure 12 (i.e., the stroke presence/absence and kind were always correctly classified), while SVM had a lower correctness percentage for the healthy cases. Within the ischemic and haemorrhagic macro-classes, there were some errors in the stroke location, mainly due to ambiguous positions where the (randomly chosen) stroke centre was close to the axis dividing two head regions.

As shown in Figure 13b,e, the MLP results for System B were identical to the ones for System A, never missing the macro-classes. Instead, SVM and k-NN were worse in the classification of healthy cases. In particular, k-NN was sensitive to the system change: with System A, it never missed the macro-classes, while with System B, it classified three healthy scenarios as ischemic stroke, as reported Figure 13c,f.

Analysing the simulation times, MLP’s training phase was the longest one (580 s for MLP, 10 s for SVM, and 0.6 s for k-NN), while its testing prediction time was the shortest one. In the considered classification of 125 samples, the testing prediction time was 0.04 s for MLP, 0.21 s for SVM, and 3.32 s for k-NN.

### 5.2. Amplitude Dataset

**Figure 14:**
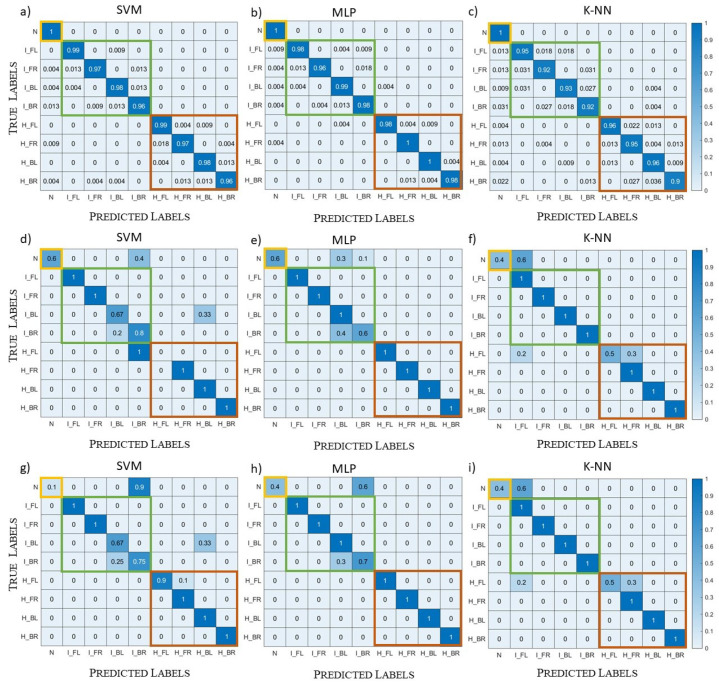
Normalized confusion matrices, amplitude dataset: (**a**–**c**) validation; (**d**–**f**) System A testing; (**g**–**i**) System B testing; (**a**,**d**,**g**) SVM results; (**b**,**e**,**h**) MLP results; (**c**,**f**,**i**) k-NN results.

Here, we investigate the possibility to limit the dataset, in the input to the ML algorithms, to its amplitude only, i.e., discarding the phase of the scattering parameters. This analysis can be of interest considering the lower cost and higher simplicity of a receiving system that measures amplitude only. The grid search was used again to select the best set of hyperparameters. They were very close to the ones used for the complex datasets, except for the kernel coefficient γ in SVM, which was equal to 0.11, and the learning rate used for MLP, which in this case is the constant one. Figure 14 reports the normalized confusion matrices for the validation (first row), the testing with System A (second row), and the testing with System B (third row). In the validation, the performances of the three algorithms were comparable to the ones in Section 5.1: the percentage of the total number of incorrect classifications for SVM was 2.5%, for MLP was 1.5%, and for k-NN was 5.6% (see Figure 14a–c). However, in the testing cases (see Figure 14d–i), the algorithms’ performances were worse, and, in particular, they had difficulties in the classification of the healthy scenario, while there were some cases of mistakes in the stroke type classification. This can be due to the fact that the phase gives information about the stroke position and, in a multi-tissue head, the dielectric contrast is a function of the position: an ischemic stroke in a tissue can have the same dielectric contrast of a haemorrhagic stroke in a different tissue. Moreover, keeping the amplitude only, the features were reduced by a factor two and a larger training set could be necessary.

### 5.3. Different Head Models’ Datasets

In this section, we analyse the performance of the algorithms when new head models are introduced. In particular, we exploited the same mechanism to create the differential scattering parameters described in Section 4.2, with only one linearized integral operator corresponding to the scenario with the original head model. As in the previous test, the features of the dataset were the total scattering parameters ($S^{t}$), so we had to sum the differential scattering parameters (ΔS) to the background scattering parameters ($S^{b}$), but in this case, we considered more than one head model and, therefore, more than one $S^{b}$. The values of ΔS were summed to different $S^{b}$, distributing them equally among the models.

**Figure 15:**
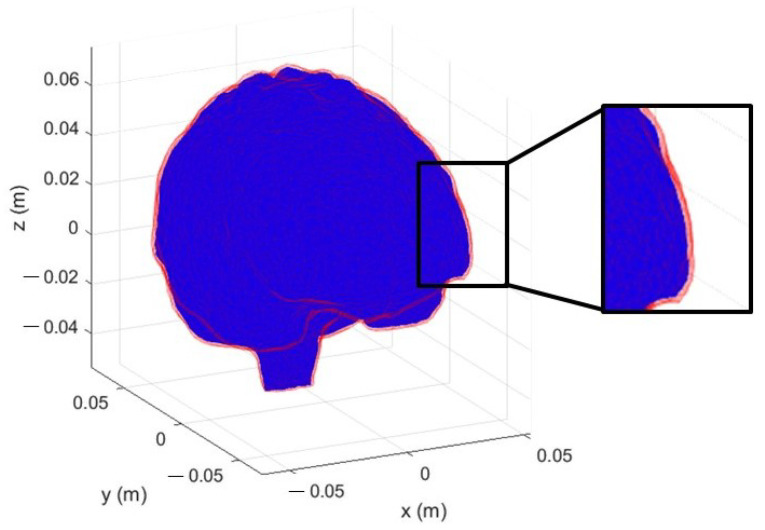
Grey matter volume variation: starting volume in blue; layer of variation in red.

Starting from the head shown in Figure 2, we modified the tissues’ boundaries simulating not only new heads, but also possible movements of the same head in the system. In this process, we employed always the same discretization of the head volume, without the need to have new CAD head models, because the tetrahedral cells already allow efficiently modifying the original head, giving considerable flexibility in the creation of new models. In particular, after the identification of the tetrahedra pairs with a face in common and belonging to different tissues, we expanded a chosen tissue, just assigning its dielectric properties to the boundary’s tetrahedra of the other one. This method can be applied iteratively to expand or reduce each tissue layer by layer. In Figure 15, we report an example of 6 mm boundaries’ modification of the grey matter, which corresponds to a change of the dielectric properties of the two layers of the tetrahedra at the tissue interface (each layer of tetrahedra has a thickness of around 3 mm).

Moreover, in the head model exploited so far, we assigned at each tetrahedron belonging to a tissue the same dielectric properties, but in a biological tissue, the properties cannot be perfectly homogeneous. For this reason, a method to make the head more realistic is to add a variation separately for each tissue of the head: choosing a percentage of variation var, the permittivity and conductivity of each tissue have a range between −var% and +var% of their nominal values.

In order to generalize the training set, we created five different head models, by moving the tissues’ boundaries for two tetrahedra layers and with different combinations. For each created model, we obtained two models by adding two different percentages of the dielectric properties’ variation in the tissues to eliminate the unrealistic tissues’ homogeneity: ±0.3% and ±5.0% with respect to the nominal dielectric properties’ values. Now, we had five head models, each one with two different percentage variations of the tissues, for a total of 10 models. From these 10 models, we calculated 10 background scattering parameters ($S_{i}^{b}$ with i=1,…,10) through full-wave simulations with the FEM solver. Once having created 10,000 ΔS with the integral linearized operator as explained in Section 4.2, we added the 10 $S_{i}^{b}$ parameters in an equally distributed way to the dataset: each model had 1000 samples.

For each algorithm, the set of hyperparameters was selected again through the grid search method. In SVM, the kernel used was the radial basis function with the kernel coefficient γ equal to 0.059 and the penalty parameter *C* equal to 300. In MLP, there were five hidden layers with 800, 400, 200, 100, and 50 neurons each, the hyperbolic tangent was the activation function, the solver was the stochastic gradient descent, and the regularization term α was equal to 0.0001. The maximum number of epochs was 1000, and the size of the batch was equal to 200; finally, the learning rate was constant. In k-NN, we used the Euclidean distance metric, with *k* equal to 3.

**Figure 16:**
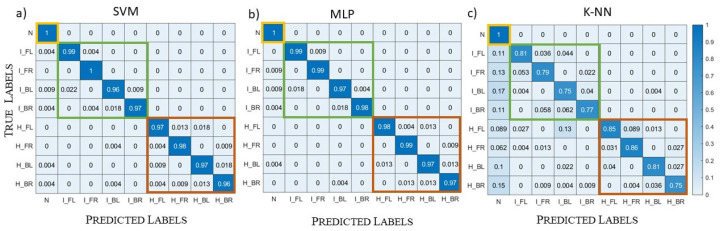
Validation case, complex datasets; normalized confusion matrices: (**a**) SVM results; (**b**) MLP results; (**c**) k-NN results.

As in the previous sections, we divided the dataset into a training set (80% of the dataset) and a validation set (20% of the dataset). Here, we report only the results of the complex dataset. The results of the validation, shown in Figure 16, were very similar to the ones obtained in Section 5.1 for the SVM and MLP algorithms and a bit worse for the k-NN one.

The next step was the testing set generation, in which we included not only the models of the training set, but also intermediate models with one layer of tetrahedra variation with respect to the original model, for a total of nine different models. For all these models, we created the healthy, the haemorrhagic stroke, and the ischemic stroke case, for a total of 27 samples. Moreover, we eliminated the tissues’ homogeneity with a variation of 2.0%, intermediate with respect to the ones of the training set. SVM and MLP correctly classified all the samples in the testing set, so we can deduce that these two algorithms learned to generalize. Instead, k-NN reached an accuracy equal to 67%, probably because this kind of algorithm has fewer hyperparameters and, therefore, less flexibility if compared with the other two. Moreover, the value of *k* selected by the grid search method was very small, and this does not help in a dataset with a high variability of the samples in each class.

## 6. Conclusions and Perspectives

In this paper, machine learning algorithms were applied to a microwave imaging system in order to classify brain stroke. One drawback in the use of ML algorithms is the need for a large amount of data in the training phase, which, in this application, can be not easily obtained or its generation needs a significant time and effort. We proposed an alternative and efficient method to create the training dataset, based on the distorted Born approximation, to obtain a linear scattering operator from the dielectric contrast space to the scattering parameters’ one. To generate a dataset of 10,000 examples, the simulation time, via a non-optimized Matlab code, was equal to 13 h with respect to 3.5 years with the standard FEM full-wave simulations.

To analyse the ML algorithms’ performance, validation and testing were performed also considering MWI systems slightly different from the nominal one, as could happen in a real scenario. We tested three different ML algorithms: SVM, MLP, and k-NN. The algorithm with the best performance was MLP, which, in particular in the testing phase with complex input records, never mistook the macro-classes, i.e., always identifying correctly the stroke presence/absence and type. If the dataset was limited to amplitude only, the ML algorithms’ performance was worse, probably also because the reduction of the features would need a larger training set.

Moreover, a further test was performed, with the introduction of more head models in the training set. This analysis aimed to understand if the algorithms were able to generalize and classify also examples with a different head model. The testing set, in this case, comprised also samples with head models different from the ones exploited in the training set. From the results, we saw that MLP and SVM correctly classified all the testing set, while k-NN proved that it was unable to generalize, reaching a low accuracy.

The next steps are further investigations into using this approach by introducing more cases in the testing set and the ML performance also with measured scattering parameters, e.g., with the MWI system described in [15] applied to 3D multi-tissue anthropomorphic head phantoms.

## Data Availability

Not applicable.

## Abbreviations

The following abbreviations are used in this manuscript:

MRI	Magnetic resonance imaging
CT	Computerized tomography
MWI	Microwave imaging
ML	Machine learning
EM	Electromagnetic
SVM	Support vector machine
MLP	Multilayer perceptron
k-NN	k-nearest neighbours
DoI	Domain of interest
FEM	Finite element method
